# Green Worship: The Effects of Devotional and Behavioral Factors on Adopting Electronic Incense Products in Religious Practices

**DOI:** 10.3390/ijerph16193618

**Published:** 2019-09-26

**Authors:** Zhenzhen Qin, Yao Song, Yang Jin

**Affiliations:** 1 School of Journalism and Communication, Anhui Normal University, Wuhu 241002, China; 2 School of Design, The Hong Kong Polytechnic University, Hung Hom, Hong Kong 00852, China; 3 General Affairs Department, Anhui College of Traditional Chinese Medicine, Wuhu 241002, China; jinyang@ahzyygz.edu.cn

**Keywords:** health-promotion behavior, Buddhist practices, cultural product design, self-efficacy, religiosity, public policy for incense smoke

## Abstract

The Buddhist tradition of incense burning has been practiced in many Asian countries. Prior studies have indicated that frequent exposure to incense smoke is linked to carcinogen-related health issues. However, widespread acceptance of health-friendly electronic incense and rituals remains limited. Based on theories of religious perceptions and health behavior, the present study aims to identify the factors which influence the acceptance of electronic incense burning in religious practices. A between-subjects (105 Buddhist participants) experiment was designed to compare the effects of different incense burners on religiosity, worship intention, perceived self-efficacy, health-promotion intention, and its underlying mechanism. Our results imply that Buddhists tend to show similar religiosity and worship intention in three different scenarios, namely, the usage of a traditional incense burner, an electronic incense burner, and an electronic burner attached with a doctrine reminder. Buddhists also tend to have a higher perceived self-efficacy and higher health-promotion intention when exposed to electronic incense scenarios (either with or without the attached doctrine reminder). The perceived self-efficacy was found to mediate the effect of the incense burning on health-promotion intention. Important implications for public policies are also discussed.

## 1. Introduction

The Buddhist tradition of incense burning has been practiced in many Asian countries, such as China, Thailand, and Korea [1,2]. Its odor and smoke have been regarded as an essential way to sanctify the ritualistic behavior in Buddhist practices. With the rising awareness of health issues, nevertheless, studies have indicated that exposure to incense smoke may be linked to carcinogen-related health issues [3,4,5,6,7,8,9,10,11,12]. Though many scholars have exerted efforts to reduce health risks involved in incense burning, such as smoke emission reduction [6,13] and ventilation conditions improvement [14], the behavioral interventions have received little research attention.

With the rapid development of alternative electronic products that reduce health-threatening behaviors [15,16,17], various health-friendly incense products have been designed and promoted to replace the traditional incense burning at temples and other indoor places [18,19]. Such an electronic simulation of incense burning can decrease threats to health; however, its widespread acceptance remains limited [20]. Although it has been suggested that religiosity is the main reason people feel reluctant to use electronic incense products [21], few studies have been conducted. According to Mamudu and Studlar’s relevant studies [22,23,24,25] on tobacco control, the understanding of individuals’ psychological factors is useful in facilitating relevant policy-making. Given this, an investigation of psychological factors associated with Buddhist incense burning could help practitioners to promote wider acceptance of electronic incense burning in religious practices.

Based on theories of religious perceptions and health behavior, the present study aims to identify the factors which influence the acceptance of electronic incense burning in religious practices. Relevant managerial implications are also discussed to promote health-friendly incense products and future practices.

## 2. Literature Review

### 2.1. Incense Burning Related Health Issues and Electronic Incense Products

Worshipping Buddhist divine beings through burning incense sticks has been viewed as an important ritualistic practice in Asian nations for centuries [1]. Previous research shows that approximately half of the populations across Southeast Asia burn incense at home as a daily ritual [2]. Buddhists regard incense as “divine odor,” and its smell is meant to evoke the presence of Buddhist divinities. Burning incense sticks works as a sensory way to sanctify the space and offering behaviors with mindfulness and awareness [26,27,28]. During the ritual process, an individual waves three or more burned incense sticks overhead while bowing to the divine statues. The burning incense sticks are then vertically placed into a censer located in front of the statues. To date, incense burning has become an indispensable ritualistic practice for many people who believe in Buddhism. Previous literature suggests that similar rituals of incense burning are also practiced in other major religions, such as Taoism [29] and Hinduism [30].

In 2003, the Environmental Protection Agency in Taiwan reported that a total of 28.7 metric tons of incense were burned in 92 temples in Kao-Hsiong City, which contributes to approximately 3580 tons of incense consumed per year [2]. Even larger quantities of incenses are burned during religious festivals, such as Buddha’s Birthday [28]. Moreover, due to the recent rapid development and prevalence of religious tourism in Asian regions, the ever-increasing number of visitors has continually promoted the consumption of incense at temples and other religious sites.

Incense smoke has been known to threaten public health since the late 1960s [31]. Conventionally, Buddhist incenses are made from aromatic biotic material that emits fragrant smoke when burned [32]. The incense smoke emitted during the burning process is a complex mixture of gases and particles, which contain a multitude of possible carcinogens, including polycyclic aromatic hydrocarbons (PAHs) [3], carbonyls [4], aliphatic aldehydes [5], and benzene [8]. Recent studies have revealed that a number of adverse health effects, such as risks of childhood brain tumors (odds ratio = 3.3, *p* = 0.005) [9], asthma (*p* = 0.05) [33], dermatitis [10], hypertension [34], and other health impacts [11] are related to frequent exposure to incense smoke. For instance, scholars [35] have preliminarily indicated that exposure to carcinogens emitted from incense burning may increase the risk of cancer in temple workers more than control workers. An increased risk of cardiovascular mortality is associated with long-term exposure to incense burning in the indoor environment [36]. Furthermore, a large number of inferior incenses are revealed using chemical fragrance, such as sawdust, industrial resins, and flavors to reduce costs. Its potential health effects have also raised scholars’ concerns [37,38].

In this context, relevant studies have investigated various approaches to incense-related health issues, such as smoke emission reduction [6,13] and ventilation conditions improvement [14]. Though recent scholars have studied the public actions of risk behavior reduction (e.g., tobacco control) [2,39,40], the individual’s perceptual reaction to incense burning receives little attention, which makes it difficult to determine relevant policies.

Alternative electronic solutions, such as electronic cigarettes [15,16,17], are introduced and adopted to intervene in health-threatening behaviors. With fewer health issues, they tend to compensate for people’s psychological needs [41]. Through the usage of electronic simulation of incense burning, people might have similar religious experiences under the condition of retaining the original burning process, setting up a bridge between traditional Buddhism and a healthy life [42]. In this view, various health-friendly electronic incense products have been designed and developed, aiming to replace traditional incense burning at temples and other indoor places [18,19]. After reviewing most incense products and relevant patents on the market, the current electronic incense products can be divided into three main categories: (1) simply using plastic and ceramic materials to simulate the visual appearance of traditional incense sticks and burners with burning-like electric lights—without any fire, smoke, and aroma—which is the healthiest design solution only involving visual appearance; and (2) in addition to the visual appearance simulation of incense stick and burner (heat-resisting materials, such as ceramics), some electronic incense burners take advantage of electric conduction to emit aroma instead of burning(the aromatic materials used are the same with traditional incense), thus, there is no smoke and particles—the main reason leading to adverse health effects—emitted during this process [43], a comparison of released smoke between heat-not-burn aroma products and traditional ones has been studied [44]; and (3) except for the visual appearance simulation of incense stick and burner, using a perfume vaporizer to simulate the burning-like smoke and aroma emitted by traditional incense, such a perfume vaporizer, which uses a heating element to vaporize a solution for provision without burning process and smoke emitting, which is the same technical patent adopted in most electric air diffusers and humidifiers [45]. The third category is more experiential and relatively health-friendly than the previous two categories. Its health effects are mainly decided by the perfume (or essential plant oils) used (their qualities are controlled by national food and drug administration departments). Though more and more governments and religious institutions advocate health-promotion behaviors in religious practices, electronic incense products and rituals are still seldom adopted [20].

Similar to other religions, Buddhism welcome scientific discoveries [46]. Also, relevant research has indicated that Buddhists have long embraced the spirit of science [47]. Buddhism and Buddhists are open-minded to accept scientific principles or technology innovations [46,47,48]. For example, “Heart Sincerity Buddha Bless” (“心诚则灵” in Chinese) is a famous Buddhist doctrine, suggesting that sincere worship does not need to care about the ways or tools used [47,48]. Therefore, Buddhist doctrines do not refuse technology; instead, they might embrace changes in their practice [46].

It has been suggested that religiosity is the primary reason people feel reluctant to use electronic incense products [21], since keeping the traditional way of Buddhist rituals is regarded as necessary in shaping religiosity. Nevertheless, the latest research has argued that technology and science might facilitate religious practices, promote spirituality, and transform concepts of religion and myth [49,50,51,52]. Accordingly, the reason why electronic incense lacks large-scale adoption might not lie in the fact that Buddhism and Buddhists are reluctant to accept new technology in their religious practices [53,54]. An investigation of the psychological patterns of incense burning can help practitioners to generate the managerial implications for reducing traditional incense burning.

### 2.2. Religiosity and Worship Intention through Incense Burning

Perceived religiosity has been widely adopted in relevant social studies to predict consequent religion-oriented behaviors [55,56,57]. According to Glock and Stark [58], five dimensions, namely experiential, ritualistic, ideological, intellectual, and consequential, have been identified to describe the concept of religiosity. Among those, the ritualistic domain involves the worship experience within a religious community and sites, such as temples. Allport and Ross [59] have further concluded that religiosity is a two-dimensional intrinsic and extrinsic construct. Intrinsic religiosity refers to a belief that religion is the framework for one’s whole life, while extrinsic religiosity refers to a self-serving and utilitarian view of religion. Those preliminary definitions of religiosity provide an understanding that religious beliefs involve a cognitive evaluation of devotion and fulfillment through ritualistic practices [60]. Given this, Buddhist worship aiming to improve and fulfill self-image [61,62] is associated with the degree of religiosity.

The effects of incense for worship, nevertheless, are ambiguous in influencing the degree of religiosity. Although religiosity stays to a certain level, in the long run, previous scholars have reported that religiosity can be increased temporarily [63,64,65]. Yeung and Chow [66] suggest that more engagement in Buddhist practices would strengthen adolescents’ religiosity. Hence, it can be inferred that Buddhists’ religiosity is influenced by worship practices. If an individual presents a similar degree of religiosity whether facing a traditional incense and an electronic one, it helps to exclude the previous concern of religiosity for the reluctance of electronic incense products.

Also, an individual’s worship intention influenced by a health-friendly electronic incense product is unclear in previous literature. It is helpful to empirically investigate whether an individual’s intention to worship is influenced by the physical tools he or she adopted.

### 2.3. Health Behavior Theories

According to the widely accepted theory of reasoned action [67], the stronger the motivated intention to engage in a behavior, the more likely its performance. Attitude, influenced by the evaluation of the behavior and subjective norm, indicates the degree of intentions to perform the behavior. This model has been applied to health-related studies of the relations among beliefs, attitudes, behavioral intentions, and behaviors. Previous literature suggests that an individual’s health-promotion behavior is predicted by attitudinal intention. However, the importance of these constructs relative to one another is likely influenced by cultural differences, which means the intentions to perform behaviors are more possibly predicted by attitudes, rather than subjective norms [68,69].

Albert Bandura, later, argues that an individual’s motivation to perform a behavior is affected by the personal judgment of “how well one can execute courses of action required to deal with prospective situations” [64]. The issues addressed in this line of inquiry mediate the relationship between knowledge and action. That explains why people often fail to behave optimally, even though they know full well what to do and are motivated to do it. In this view, the factor of behavioral control, known as perceived self-efficacy, has been considered to improve the predictive power of the reasoned action. Self-beliefs of efficacy, referring to the degree of confidence in the ability to take action, is useful in the self-regulation of motivation [65]. In other words, high self-efficacy leads to sufficient effort to execute a behavior, whereas low self-efficacy is likely to cease effort early [66]. The behavior control of self-efficacy contributes to mediate the psychological path of behavior performance.

Another relevant theory of the Health Belief Model [70] also suggests that an individual’s health behavior decisions are made through a computational evaluation of health-related actions and their perceived self-efficacy. Relevant studies on public health, like condom use [71] and smoking cessation [72], have applied this model to predict health-promoting behavior. Thus, it is anticipated that the degree of self-efficacy can be an effective mediator influencing an individual’s intention to adopt religious practices with electronic incense products. In addition, studies construe that self-efficacy is predicted by multiple temporary determinants, such as communicative concerns (e.g., verbal persuasion) and task environment [73,74]. Based on these findings, we assume that the incense could work as a physical cue to remind people of relevant doctrines in Buddhism (e.g., “Heart Sincerity Buddha Bless” in Section 2.1), and then enhance the perceptions of self-efficacy.

## 3. Hypotheses Development

Based on the literature in Section 2, we propose that people might present similar religiosity and worship intention towards electronic incense products and the traditional ones. Also, electronic incense products might significantly improve health-promotion intention, and this process might be mediated by the perceived self-efficacy. Accordingly, we hypothesized as follows:

**Hypothesis** **1** **(H1).** *People would hold similar religiosity towards electronic incense products and the traditional incense burner.*

**Hypothesis** **2** **(H2).** *People would hold similar worship intention towards electronic incense products and the traditional incense burner.*

**Hypothesis** **3** **(H3).** *Electronic incense products would elicit higher self-efficacy in religious practices compared with the traditional incense burner.*

**Hypothesis** **4** **(H4).** *Electronic incense products would elicit a higher health-promotion intention in religious practices compared with the traditional incense burner.*

**Hypothesis** **5** **(H5).** *The effect of an incense burner on health promotion behavior intention in religious practices is mediated by perceived self-efficacy.*

## 4. Methods

### 4.1. Measurement Items

As for the measurement items, we adopted a nine-point Likert scale to measure the factors mentioned before. All the measurement items or scales were retrieved from the relevant research. To be more specific, the Buddhism religiosity was measured by a five-item scale in previous research (sample item “Buddhism forms an important basis for the kind of person I want to be”; 1 = Strongly Disagree, 9 = Strongly Agree; Cronbach’s alpha was 0.933) [75]. The worship intention was estimated by three items in Dodds, Mon, and Grewal’s work on behavioral intentions (sample item “I am willing to use this incense to pray”; 1 = Strongly Disagree, 9 = Strongly Agree; Cronbach’s alpha was 0.952) [76]. Besides, a three-item scale from Kim and Choi’s work measured self-efficacy on health (sample item “My behavior can have a positive effect in support of promoting health”; 1 = Strongly Disagree, 9 = Strongly Agree; Cronbach’s alpha was 0.724) [77]. Following Filipkowski et al.’s work and suggestions [78], the health-promotion practice was estimated by a single-item scale, which has been proven to be robust and reliable in terms of estimating health-promotion intention (measurement item “The probability for me to adopt health-promotion behavior is high”; 1 = Strongly Disagree, 9 = Strongly Agree) [79]. The Cronbach’s alpha of all the constructs is above 0.7, suggesting that the items are consistent and reliable in the current study. The whole measurements could be found in Table 1.

### 4.2. Experiment Stimuli

**Figure 1:**
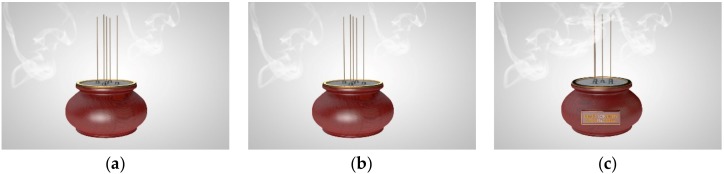
(**a**) Traditional Burner, (**b**) Electronic Burner, and (**c**) Electronic Burner with doctrine reminder.

Concerning the experiment stimuli and design, we deployed a between-subjects experiment to examine Hypotheses 1–5. To specify, three burner scenarios were designed: A traditional burner, an electronic burner, and an electronic burner with semantic reminding. We recruited a professional product designer to 3D-model these scenarios (see Figure 1a–c).

In order to control the confounding factors in the designed stimuli, the designer carefully made the burner with the same height, width, size, etc. To be more specific, the Figure 1a scenario was described with the statement, “It is a traditional incense in Buddhist rituals. As you might notice, traditional incense could emit smoke in the air”. The Figure 1b scenario was described with the statements, “It is a health-friendly electronic incense using a light-emitting diode (LED), coating materials, and safe perfume vaporizer to simulate the burning-like appearance and aroma emitted by traditional incense sticks”. The Figure 1c scenario was described with the statements, “It is a health-friendly electronic incense using a light-emitting diode (LED), coating materials, and safe perfume vaporizer to simulate the burning-like appearance and aroma emitted by traditional incense sticks. Also, Buddhism advocates, ‘Heart Sincerity Buddha Bless’, suggesting Buddhists can pray without concerning the tool he or she used to pray”. Accordingly, we made sure that the differences among these three scenarios were the health-friendly technology function and the semantic reminder.

### 4.3. Experiment Process

We recruited participants via Amazon Mechanical Turk (AMT). AMT is a valid data collection source with over 500,000 workers all around the world and many health-related, mental, or medical experiments have been conducted through it [80,81,82]. It has been proved to have sufficient quality and reliability when compared with experimental data collected in physical laboratories [83]. One of the most significant advantages to adopting AMT lies in that it could recruit workers from various regions and countries, not only avoiding snowball sampling but also representing the Buddhist population to a larger degree [84]. Thus, we decided to use this platform to recruit our participants.

The reference number of the ethical approval for research involving human subjects in Anhui College of Traditional Chinese Medicine is HSERS2019050001. Firstly, we created the questionnaire with Qualtrics, a prevalent web-based survey software. Then, we published our task in AMT with specific requirements (e.g., used to practice Buddhist incense burning) and a link to the questionnaire. Participants browsed the AMT website, interested in participating, checked the criteria, could click “Accept the Task”, and could then be directed to the questionnaire prepared. As a result, a total of 105 Buddhist participants were recruited (mean age = 34.48 years; 64 men and 41 women) to take part in this study. The structure of the questionnaire included several parts: (1) a short introduction describing the procedure of the experiment; (2) the informed consent (participants consent to us recording their key-press responses via AMT, the data would be kept completely confidential and anonymous, and they were informed that they could withdraw at any time if they felt uncomfortable); (3) personal characteristics (age, gender, etc.); (4) the main experiment as described in Section 4.2, all participants were randomly divided into three groups (each group contained 35 participants and they were required to carefully read the descriptions and evaluate the incense burner based on the questions given); (5) a single-item manipulation check (people have to choose the extent they agree on a statement, “The emission from this incense would not harm my health”); and (6) participants submitted their responses and were informed that they had finished the experiment.

## 5. Findings

We used SPSS 22.0 (IBM Corporation, New York, NY, USA) to investigate the data in this study. There were no missing or incomplete responses. To begin with, we checked the manipulation check, indicating a significant difference between the three scenarios. According to the result of one-way ANOVA, people who were exposed to the electronic incense burner with or without reminding of Buddhist doctrine (Mean = 7.43 and 7.37, respectively) rated significantly higher than those exposed to the traditional burner (Mean = 3.94; F(2, 102) = 37.18, *p* < 0.01). It suggested that our manipulation was successful. Besides this, the skewness and kurtosis of all the constructs were also examined. While skewness estimates symmetry of the data set, kurtosis examines whether the data set is heavy-tailed compared with normal distribution [85]. Results indicated the skewness and kurtosis are all within the threshold, skewness ranged from −1.154 to −0.054, and the kurtosis ranged from −1.10 to 0.340, respectively [85,86]. Accordingly, the current dataset generally followed a normal distribution. Table 2 shows the means, SD (standard deviations), and Pearson correlations of the different factors.

**Figure 2:**
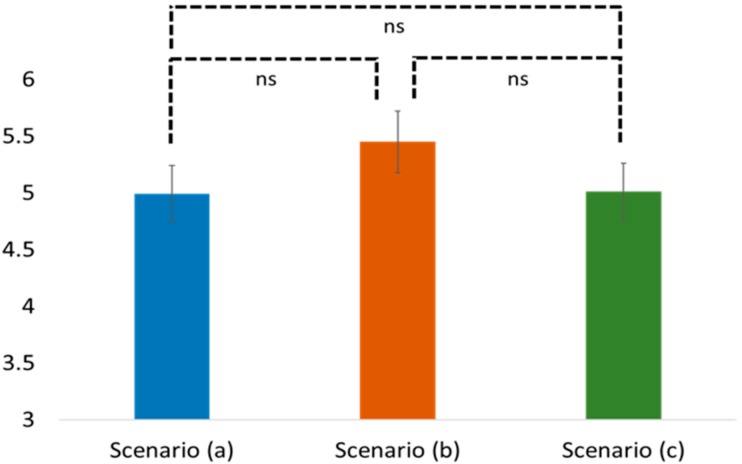
Comparison of religiosity in different scenarios. Note: ns stands for non-significant.

**Figure 3:**
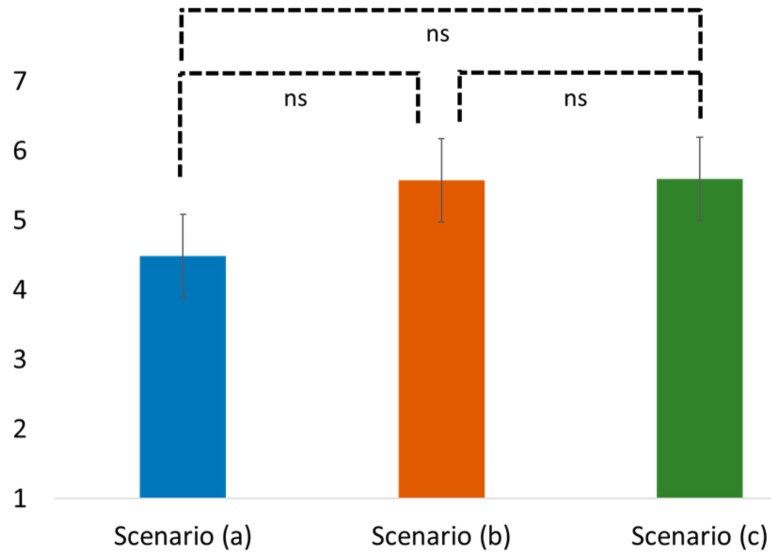
Comparison of worship intention in different scenarios. Note: ns stands for non-significant.

Testing for H1 and H2, one-way ANOVA, and post-hoc Tukey HSD were performed with different incense burners (a traditional burner vs. an electronic burner vs. an electronic burner with doctrine reminder) as the predictor variables, and the perceived religiosity and worship intention as the predicted variables. According to the results of the one-way ANOVA, we found an insignificant difference between the perceived religiosity and worship intention among these three scenarios (religiosity: F(2, 102) = 0.439, *p* = 0.646; worship intention: F(2, 102) = 2.115, *p* = 0.126; see Figure 2 and Figure 3).

Particularly, the post-hoc Tukey HSD results revealed that people in the traditional incense burner scenario (a) showed similar perceived religiosity and worship intention when compared with the electronic incense burner (b) (Tukey HSD, religiosity: Mean = 4.99 vs. 5.45; SD = 2.10 vs. 2.33; *p* = 0.688; worship: Mean = 4.49 vs. 5.58; SD = 2.66 vs. 2.38; *p* = 0.186). Similar observations were also found between scenarios (a) and (c) (Tukey HSD, religiosity: Mean = 4.99 vs. 5.01; SD = 2.10 vs. 2.48; *p* = 0.999; worship: Mean = 4.49 vs. 5.60; SD = 2.66 vs. 2.65; *p* = 0.176) and between scenarios (b) and (c) (Tukey HSD, religiosity: Mean = 5.45 vs. 5.01; SD = 2.33 vs. 2.48; *p* = 0.707; worship: Mean = 5.58 vs. 5.60; SD = 2.38 vs. 2.65; *p* = 0.999). Thus, both H1 and H2 were supported.

**Figure 4:**
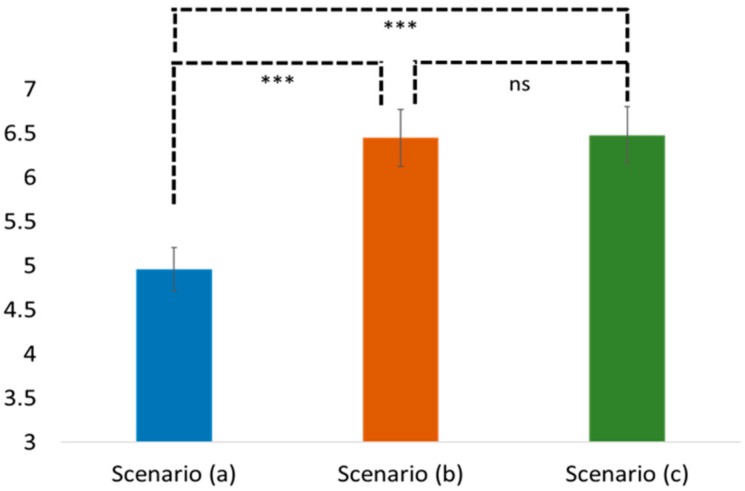
Comparison of the perceived self-efficacy in different scenarios. Note: ns stands for non-significant; *** means < 0.01.

**Figure 5:**
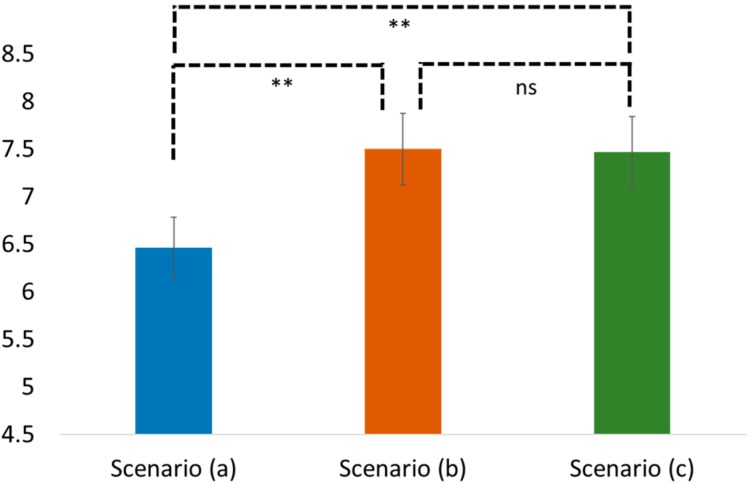
Comparison of the health-friendly Buddhist practice in different scenarios. Note: ns stands for non-significant; ** means < 0.05.

Regarding H3 and H4, the results of the one-way ANOVA similarly showed a significant difference in the perceived self-efficacy and the health-promotion intention among these three scenarios (the perceived self-efficacy: F(2, 102) = 9.169, *p* < 0.05; the health-promotion intention: F(2, 102) = 11.695, *p* < 0.05; see Figure 4 and Figure 5). The post-hoc Tukey HSD results showed that people in scenario (b) showed significantly higher perceived self-efficacy and health-promotion intention than those in scenario (a) (Tukey HSD, self-efficacy: Mean = 6.45 vs. 4.94; SD = 1.41 vs. 1.85; *p* < 0.05 and health-promotion: Mean = 7.54 vs. 6.57; SD = 1.12 vs. 1.91; *p* < 0.05, respectively). People in scenario (c) also showed significantly higher perceived self-efficacy and perceived health-promotion intention than those in scenario (a) (Tukey HSD, self-efficacy: Mean = 6.48 vs. 4.94; SD = 1.86 vs. 1.85; *p* < 0.05 and health-promotion: Mean = 7.60 vs. 6.57; SD = 1.31 vs. 1.91; *p* < 0.05), there was no significant difference between scenario (b) and scenario (c) in terms of the self-efficacy and the health-promotion intention (Tukey HSD, self-efficacy: Mean: 6.45 vs 6.48; SD = 1.41 vs. 1.86; *p* = 0.997 and health-promotion: Mean = 7.54 vs. 7.60; SD = 1.12 vs. 1.31; *p* = 0.996). Accordingly, H3 and H4 were supported.

**Figure 6:**
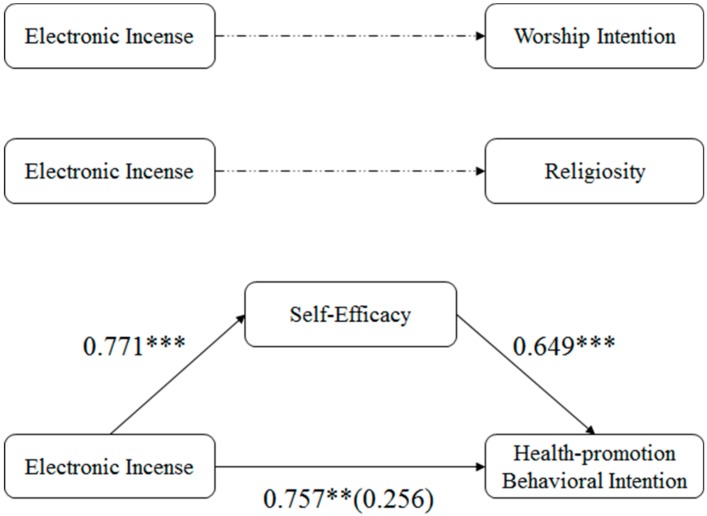
All psychological paths revealed in this study. Note: Dashed line means insignificance; solid line means significance; ** means *p* < 0.05; *** means *p* < 0.01.

In order to test H5, namely examining the mediating role of self-efficacy, we conducted PROCESS SPSS macro to validate the effect of incense burning on the health-promotion intention [87] (Model 4, n = 5000 resamples). Indeed, there are several ways to test the mediation effect. The reason why we did not use a structural equation model or path analysis [88] lies in that they overly emphasize different variables in the model, rather than treating the model as a whole. Developed by Hayes [89], PROCESS is a popular SPSS “macro” which simplifies the computational procedure of mediation, moderation, and so on in numerous behavioral studies [90,91,92]. It was believed to be a statistical method focusing on analyzing the indirect effect and the structure as a whole [93]. Thus, we believe the PROCESS could give a non-biased mediation estimation in the current study. As for the total effect, incense burning significantly improved the health-promotion intention (β = 0.757, SE = 0.319, *p* < 0.05). As for the indirect effect, incense burning positively increased the perceived self-efficacy (β = 0.771, SE = 0.209, *p* < 0.05), and the perceived self-efficacy worked as a significant predictor for the health-promotion intention (β = 0.757, SE = 0.319, *p* < 0.05). When the perceived self-efficacy was controlled, the direct effect of incense burning on the health-promotion intention was not significant (β = 0.256, SE = 0.309, *p* = 0.41). The indirect effect of incense burning on the health-promotion intention (LLCI = 0.189 and ULCI = 0.894) does not contain zero, suggesting that the mediating effect of the perceived self-efficacy was significant. Figure 6 shows all psychological paths revealed in this study. LLCI and ULCI represent lower confidence interval bound and upper confidence interval bound, respectively.

## 6. Limitation

There are some limitations worth noting. First of all, this study mainly examines the psychological factors associated with incense burning. Although previous research has validated the relationship between perceptual intention and actual behavior [94], it might be interesting to collect evidence of actual health-promotion behavior with physical electronic incense burners. Second, although all the items in the current study are adapted from the relevant literature in Table 1, some items might be confusing for the readers since there are no specific scales for the constructs. For example, the item “I could promote health by using health-friendly electronic incense” might be misunderstood, because people might be confused on the subject referred in this statement. Thus, it might theoretically be helpful to develop the related scales on self-efficacy in future research, because it is seldom discussed in the prior research [69]. Third, the cultural difference of incense burning needs to be considered in future studies. Previous studies show that Buddhist practices differ across regions [95,96]. Thus, a comparison of Buddhist responses from different regions will help to solve this issue. Last, although few negative issues of electronic incense burning have been reported [21], its potential effects in long-time usage need further investigation.

## 7. Conclusions

With fewer health threats, electronic incense products with health-friendly functions might compensate Buddhists for their ritualistic and psychological needs. However, widespread adoption and practices are still limited [20]. Concerning the serious health issues raised by traditional incense burning [1,9,11,34,35], the current study examines individuals’ perceptual path towards electronic incense burning under health awareness and religious consciousness, which is seldom investigated in previous research. Following the theories of religious perceptions and health behavior, the effects of incense burning (traditional vs. electronic) on the perceived religiosity, worship intention, perceived self-efficacy, health-promotion intention, and the associated underlying mechanism are revealed.

The current study contributes to the literature on health issues caused by incense burning from several perspectives. Our results respond to the nuanced relationship between Buddhism and emerging technology adoption of incense burning in previous research. Given that Buddhists’ similar religiosity and worship intentions in different scenarios, we might consider that electronic products tend to fulfill the equivalent psychological needs of religiosity in ritualistic practices. It supports the arguments that sincere worship in Buddhist practices goes beyond the ways or physical tools adopted [46,47,48], and new technology could enhance religious spirituality [49,50,51,52]. In other words, religiosity might not be the main reason for Buddhists’ reluctance to adopt electronic incense products in their worship practices. Indeed, Buddhism and Buddhists are open-minded to accept scientific discoveries or technology innovation [46,47,48]. Thus, this finding provides the preliminary evidence that, under certain circumstances, Buddhists could accept technology improvement in their religious practices even without being reminded of the doctrine of heart sincerity. The reason for the limited scale of adoption of electronic incense products might lie in other psychological factors [53,54].

In addition, Buddhists tend to have higher perceived self-efficacy and higher health-promotion intentions when exposed to electronic incense scenarios (either with or without the attached doctrine reminder), and the perceived self-efficacy works as a significant mediator in the relationship between incense burning and the health-promotion intention. This finding indicates that electronic incense products promote health consciousness during Buddhist worship. Also, the current study supports previous literature on the mediator role of self-efficacy in health-promotion behavior [97].

There are also some managerial implications for this study. Given the increasing attention to health issues associated with traditional incense burning [3,4,6,7,8,9,10,11,33,34], it is necessary to explore an effective solution to promote health-friendly products in religious practices. Electronic incense products—with health-friendly visual and aromatic stimulations of traditional incense—seem to be a relatively satisfactory solution to improve individual’s health behavior and religious spirituality. Consequently, religious institutions and governments can consider adopting relevant health policies, electronic incense products, and installations with health-friendly designs and aromatic materials on a larger scale, especially in Buddhism-dominated regions. For instance, relevant regulations of safety control of such aromatic materials should be identified by national food and drug administration departments when introducing electronic incense products. Furthermore, since self-efficacy promotes individuals’ health-promotion intentions of incense burning, relevant communicative approaches, like advertising and campaigns against traditional incense burning, should underline the individual’s abilities to achieve the goal of healthy life with religions.

## Figures and Tables

**Table 1:** Description of different variables and their measurements.

Measure	Variable Category	Measure Items	References
Incense burning	Independent Variable	Categorical coding: 1 for a traditional burner; 2 for an electronic burner; 3 for an electronic burner with doctrine reminder, as described in Figure 1	
Buddhism religiosity	Dependent Variable	Buddhism forms an important basis for the kind of person I want to be. My ideas on Buddhism have a big influence on my view in other areas. Were I to think about Buddhism differently, my whole life would be very different. I often think about Buddhist matters. Buddhism is one of the most important parts of my philosophy of life.	Putney and Middleton, 2006
Worship intention	Dependent Variable	I am willing to use this incense to pray. The likelihood for me to pray with this incense is high. Probability for me to pray with this incense is high.	Dodds, Mon and Grewal, 1991
Self-efficacy on health	Mediator	My behavior can have a positive effect in support of promoting health. I feel I could keep health by using this electronic incense. I could promote health by using health-friendly electronic incense.	Kim and Choi, 2005
Health-promotion intention	Dependent Variable	The probability for me to adopt health-promotion behavior is high	Filipkowski et al., 2010

**Table 2:** Means, standard deviations, and correlations of different constructs.

Construct	Mean	SD	RE	WI	HI	PS
Religiosity (RE)	5.15	2.23	1.000			
Worship Intention (WI)	5.22	2.60	0.532 ***	1.000		
Health-promotion intention (HI)	7.24	1.54	0.030	0.175	1.000	
Perceived Self-Efficacy (PS)	5.96	1.85	0.165	0.478 ***	0.515 ***	1.000

Note: SD means standard deviation; RE stands for Religiosity; WI stands for Worship Intention; HI stands for Health-promotion intention; PS stands for Perceived Self-Efficacy; *** means *p* < 0.01.

## References

[1] Lin T.C., Krishnaswamy G., Chi D.S. (2008). Incense smoke: Clinical, structural and molecular effects on airway disease. Clin. Mol. Allergy.

[2] Friborg J.T., Yuan J.M., Wang R., Koh W.P., Lee H.P., Yue M.C. (2008). Incense use and respiratory tract carcinomas: A prospective cohort study. Cancer.

[3] Liao C.-M., Chiang K.-C. (2006). Probabilistic risk assessment for personal exposure to carcinogenic polycyclic aromatic hydrocarbons in Taiwanese temples. Chemosphere.

[4] Ho S.S.H., Yu J.Z. (2002). Concentrations of formaldehyde and other carbonyls in environments affected by incense burning. J. Environ. Monit..

[5] Yang T.T., Chen C.C., Lin J.M. (2006). Characterization of Gas and Particle Emission from Smoldering Incenses with Various Diameters. Bull. Environ. Contam. Toxicol..

[6] Yang T.-T., Kuo Y.M., Hung H.F., Shie R.H., Chang P. (2017). Gas Pollutant Emissions from Smoldering Incense Using FTIR. Aerosol Air Qual. Res..

[7] Yang C.-R., Lin T.-C., Chang F.-H. (2007). Particle size distribution and PAH concentrations of incense smoke in a combustion chamber. Environ. Pollut..

[8] Yang T.T., Lin T.S., Chang M. (2007). Characteristics of Emissions of Volatile Organic Compounds from Smoldering Incense. Bull. Environ. Contam. Toxicol..

[9] Preston-Martin S., Yu M.C., Benton B., Henderson B.E. (1982). N-Nitroso compounds and childhood brain tumors: A case-control study. Cancer Res..

[10] Hayakawa R., Matsunaga K., Arima Y. (1987). Depigmented contact dermatitis due to incense. Contact Dermat..

[11] See S.W., Balasubramanian R., Man Joshi U. (2007). Physical characteristics of nanoparticles emitted from incense smoke. Sci. Technol. Adv. Mater..

[12] Orecchio S., Fiore M., Barreca S., Vara G., Orecchio S., Fiore M., Barreca S., Vara G. (2017). Volatile Profiles of Emissions from Different Activities Analyzed Using Canister Samplers and Gas Chromatography-Mass Spectrometry (GC/MS) Analysis: A Case Study. Int. J. Environ. Res. Public Health.

[13] Yang C.-R., Lin T.-C., Peng Y.-S., Lee S.-Z., Chang Y.-F. (2012). Reducing Air Pollutant Emissions from Burning Incense with the Addition of Calcium Carbonate. Aerosol Air Qual. Res..

[14] Lung S.C.C., Kao M.C., Hu S.C. (2003). Contribution of incense burning to indoor PM10 and particle-bound polycyclic aromatic hydrocarbons under two ventilation conditions. Indoor Air.

[15] Zhu S.H., Sun J.Y., Bonnevie E., Cummins S.E., Gamst A., Yin L., Lee M. (2014). Four hundred and sixty brands of e-cigarettes and counting: Implications for product regulation. Tob. Control.

[16] Abrams D.B., Glasser A.M., Pearson J.L., Villanti A.C., Collins L.K., Niaura R.S. (2018). Harm Minimization and Tobacco Control: Reframing Societal Views of Nicotine Use to Rapidly Save Lives. Annu. Rev. Public Health.

[17] Owusu D., Aibangbee J., Collins C., Robertson C., Wang L., Littleton M.A., Boghozian R., Casenburg V., Mamudu H.M. (2017). The Use of E-cigarettes Among School-Going Adolescents in a Predominantly Rural Environment of Central Appalachia. J. Community Health.

[18] Lee B.-H. (2014). Electronic Incense Stick. U.S. Patent.

[19] Chen P.-C., Cheng T. (2013). Electronic Incense Stick Having High Light Usage Efficiency. U.S. Patent.

[20] Wong Tai Sin, an Environmentally Friendly Incense Burner, No Longer Smog. http://std.stheadline.com/daily/article/detail/1539009/.

[21] Religion Goes Green in Taiwan Pollution Battle—Environment|The Star Online. https://www.thestar.com.my/news/environment/2016/05/09/religion-goes-green-in-taiwan-pollution-battle/.

[22] Mamudu H.M., John R.M., Veeranki S.P., Ouma A.E.O. (2013). The odd man out in Sub-Saharan Africa: Understanding the tobacco use prevalence in Madagascar. BMC Public Health.

[23] Mamudu H.M., Veeranki S.P., He Y., Dadkar S., Boone E. (2012). University personnel’s attitudes and behaviors toward the first tobacco-free campus policy in tennessee. J. Community Health.

[24] Mamudu H.M., Paul T.K., Wang L., Veeranki S.P., Panchal H.B., Alamian A., Sarnosky K., Budoff M. (2016). The effects of multiple coronary artery disease risk factors on subclinical atherosclerosis in a rural population in the United States. Prev. Med. (Baltim).

[25] Studlar D., Cairney P. (2019). Multilevel governance, public health and the regulation of food: Is tobacco control policy a model?. J. Public Health Policy.

[26] Budny P.G., Regan P.J., Riley P., Roberts A.H.N. (1991). Ritual burns—The Buddhist tradition. Burns.

[27] Heine S., Wright D.S. (2008). Zen Ritual—Studies of Zen Buddhist Theory in Practice.

[28] Staub P.O., Geck M.S., Weckerle C.S. (2011). Incense and ritual plant use in Southwest China: A case study among the Bai in Shaxi. J. Ethnobiol. Ethnomed..

[29] Hunter A., Dean K. (2007). Taoist Ritual and Popular Cults of South-East China. Sociol. Relig..

[30] Dewangan S., Chakrabarty R., Zielinska B., Pervez S. (2013). Emission of volatile organic compounds from religious and ritual activities in India. Environ. Monit. Assess..

[31] Schoental R., Glbbard S. (1967). Carcinogens in Chinese Incense Smoke. Nature.

[32] Morita K. (2006). The Book of Incense: Enjoying the Traditional Art of Japanese Scents.

[33] Wang I.-J., Tsai C.-H., Chen C.-H., Tung K.-Y., Lee Y.L. (2011). Glutathione S-transferase, incense burning and asthma in children. Eur. Respir. J..

[34] Song X., Ma W., Xu X., Liu T., Xiao J., Zeng W., Li X., Qian Z., Xu Y., Lin H. (2017). The Association of Domestic Incense Burning with Hypertension and Blood Pressure in Guangdong, China. Int. J. Environ. Res. Public Health.

[35] Navasumrit P., Arayasiri M., Hiang O.M.T., Leechawengwongs M., Promvijit J., Choonvisase S., Chantchaemsai S., Nakngam N., Mahidol C., Ruchirawat M. (2008). Potential health effects of exposure to carcinogenic compounds in incense smoke in temple workers. Chem. Biol. Interact..

[36] Pan A., Clark M.L., Ang L.W., Yu M.C., Yuan J.M., Koh W.P. (2015). Incense use and cardiovascular mortality among Chinese in Singapore: The Singapore Chinese health study. Environ. Health Perspect..

[37] Niebler J. (2017). Incense Materials. Springer Handbook of Odor.

[38] Zhang J., Zhang Y., Zhao B. (2010). Experimental Study on Air Pollution due to Incense Burning in Beijing Temples. Build. Sci..

[39] Mamudu H., Cairney P., Studlar D. (2015). Global public policy: Does the new venue for transnational tobacco control challenge the old way of doing things?. Public Adm..

[40] Studlar D.T. (2014). Cancer Prevention through Stealth: Science, Policy Advocacy, and Multilevel Governance in the Establishment of a “National Tobacco Control Regime” in the United States. J. Health Polit. Policy Law.

[41] Park S.H., Lee L., Shearston J.A., Weitzman M. (2017). Patterns of electronic cigarette use and level of psychological distress. PLoS ONE.

[42] Shi R. (2012). Design and Implementation of Low Carbon Incense System. Ph.D. Thesis.

[43] Yang Q. (1991). Healthy Incense Burner. China Patent.

[44] Zhu H., Xi H., Chai G., Zhao L., Liu S., Liu J., Liu S., Zeng S., Wang D., Lu B. (2017). Effects of heating temperature on release of smoky aerosol components from heat-not-burn tobacco products. Tab. Sci. Technol..

[45] Huang H.-Y. (2015). Essential Oil Diffuser. U.S. Patent.

[46] Kirthisinghe B.P. (1984). Buddhism and Science.

[47] McMahan D.L. (2010). Buddhism and Science: A Guide for the Perplexed. By Donald Lopez, S., Jr. J. Am. Acad. Relig..

[48] Yong A. (2005). Buddhism and Science: Breaking New Ground (review). Buddh. Christ. Stud..

[49] Kimura T. (2017). Robotics and AI in the sociology of religion: A human in imago roboticae. Soc. Compass.

[50] Muller M.J., Christiansen E., Nardi B., Dray S. (2001). Spiritual life and information technology. Commun. ACM.

[51] Bell G. (2006). No More SMS from Jesus: Ubicomp, Religion and Techno-spiritual Practices. Lecture Notes in Computer Science, Proceedings of the International Conference on Ubiquitous Computing, Orange County, CA, USA, 17–21 September 2006.

[52] Trovato G., De Saint Chamas L., Nishimura M., Paredes R., Lucho C., Huerta-Mercado A., Cuellar F. (2019). Religion and Robots: Towards the Synthesis of Two Extremes. Int. J. Soc. Robot..

[53] Aune M.B., DeMarinis V.M. (1996). Religious and Social Ritual: Interdisciplinary Explorations.

[54] Stavrova O., Fetchenhauer D., Schlösser T. (2013). Why are religious people happy? The effect of the social norm of religiosity across countries. Soc. Sci. Res..

[55] Peter C., Hill R.W.H. (1999). Measures of Religiosity.

[56] Mokhlis S. (2009). Relevancy and Measurement of Religiosity in Consumer Behavior Research. Int. Bus. Res..

[57] Young M., Denny G., Penhollow T., Palacios R., Morris D. (2015). Hiding the Word: Examining the Relationship Between a New Measure of Religiosity and Sexual Behavior. J. Relig. Health.

[58] Glock C.Y., Stark R. (1966). Religion and Society in Tension.

[59] Allport G.W., Michael Ross J. (1967). Personal Religious Orientation and Prejudice. J. Pers. Soc. Psychol..

[60] Holdcroft B.B. (2006). What is Religiosity. J. Cathol. Educ..

[61] Mair V.H., Blofeld J. (2006). Bodhisattva of Compassion: The Mystical Tradition of Kuan Yin. J. Asian Stud..

[62] Dupuis C.F. (2016). Origin Of All Religious Worship.

[63] Henrich J., Bauer M., Cassar A., Chytilová J., Purzycki B.G. (2019). War increases religiosity. Nat. Hum. Behav..

[64] Alam S.S., Mohd R., Hisham B. (2011). Is religiosity an important determinant on Muslim consumer behaviour in Malaysia?. J. Islam. Mark..

[65] Hassan Fathelrahman Mansour I., Mohammed Elzubier Diab D. (2016). The relationship between celebrities’ credibility and advertising effectiveness: The mediation role of religiosity. J. Islam. Mark..

[66] Yeung G.K.K., Chow W.Y. (2010). To take up your own responsibility: The religiosity of Buddhist adolescents in Hong Kong. Int. J. Child. Spiritual..

[67] Fishbein M., Ajzen I. (1975). Belief, Attitude, Intention, and Behavior: An Introduction to Theory and Research.

[68] Trafimow D., Finlay K.A. (1996). The Importance of Subjective Norms for a Minority of People: Between Subjects and within-Subjects Analyses. Personal. Soc. Psychol. Bull..

[69] Armitage C.J., Conner M. (2001). Efficacy of the theory of planned behaviour: A meta-analytic review. Br. J. Soc. Psychol..

[70] Janz N.K., Becker M.H. (1984). The Health Belief Model: A Decade Later. Health Educ. Q..

[71] Reid A.E., Aiken L.S. (2011). Integration of five health behaviour models: Common strengths and unique contributions to understanding condom use. Psychol. Health.

[72] DiClemente C.C. (1981). Self-efficacy and smoking cessation maintenance: A preliminary report. Cognit. Ther. Res..

[73] Gist M.E., Mitchell T.R. (1992). Self-Efficacy: A Theoretical Analysis of Its Determinants and Malleability. Acad. Manag. Rev..

[74] Margolis H., Mccabe P.P. (2006). Improving Self-Efficacy and Motivation. Interv. Sch. Clin..

[75] Putney S., Middleton R. (2006). Dimensions and Correlates of Religious Ideologies. Soc. Forces.

[76] Dodds W.B., Monroe K.B., Grewal D. (1991). Effects of Price, Brand, and Store Information on Buyers’ Product Evaluations. J. Mark. Res..

[77] Kim Y., Choi S.M. (2005). Antecedents of Green Purchase Behavior: An Examination of Collectivism, Environmental Concern, and Pce. ACR N. Am. Adv..

[78] Filipkowski K.B., Smyth J.M., Rutchick A.M., Santuzzi A.M., Adya M., Petrie K.J., Kaptein A.A. (2010). Do healthy people worry? Modern health worries, subjective health complaints, perceived health, and health care utilization. Int. J. Behav. Med..

[79] Idler E.L., Benyamini Y. (1997). Self-Rated Health and Mortality: A Review of Twenty-Seven Community Studies. J. Health Soc. Behav..

[80] Song Y., Qin Z., Yuan Q. (2019). The Impact of Eco-Label on the Young Chinese Generation: The Mediation Role of Environmental Awareness and Product Attributes in Green Purchase. Sustainability.

[81] Qin Z., Song Y., Tian Y. (2019). The Impact of Product Design with Traditional Cultural Properties (TCPs) on Consumer Behavior Through Cultural Perceptions: Evidence from the Young Chinese Generation. Sustainability.

[82] Song Y., Qin Z. (2019). Buddhists Care: Examining the Impact of Religious Elements on Reducing Discriminatory Attitudes toward People Living with HIV/AIDS. Religions.

[83] Stewart N., Ungemach C., Harris A.J.L., Bartels D.M., Paolacci G., Chandler J. (2015). The Average Laboratory Samples a Population of 7, 300 Amazon Mechanical Turk Workers The Size of the MTurk Population. Judgm. Decis. Mak..

[84] Dorić D., Nikolić-Dorić E., Jevremović V., Mališić J. (2009). On measuring skewness and kurtosis. Qual. Quant..

[85] Suki N.M. (2013). Green awareness effects on consumers’ purchasing decision: Some insights from Malaysia. Int. J. Asia Pac. Stud..

[86] Bolin J.H. (2014). Introduction to Mediation, Moderation, and Conditional Process Analysis: A Regression-Based Approach.

[87] Baron R.M., Kenny D.A. (1986). The Moderator-Mediator Variable Distinction in Social Psychological Research. Conceptual, Strategic, and Statistical Considerations. J. Pers. Soc. Psychol..

[88] Preacher K.J., Hayes A.F. (2004). SPSS and SAS procedures for estimating indirect effects in simple mediation models. Behav. Res. Methods Instrum. Comput..

[89] Shafi M., Yang Y., Khan Z., Yu A., Shafi M., Yang Y., Khan Z., Yu A. (2019). Vertical Co-operation in Creative Micro-Enterprises: A Case Study of Textile Crafts of Matiari District, Pakistan. Sustainability.

[90] Song Y., Qin Z. (2019). Towards the Beauty of Buddhism: The Development and Validation of a Buddhist Aesthetics Scale. Religions.

[91] Hayes A.F. (2015). An Index and Test of Linear Moderated Mediation. Multivar. Behav. Res..

[92] Hayes A.F., Montoya A.K., Rockwood N.J. (2017). The analysis of mechanisms and their contingencies: Process versus structural equation modeling. Australas. Mark. J..

[93] Sniehotta F.F., Scholz U., Schwarzer R. (2005). Psychology & Health Bridging the intention-behaviour gap: Planning, self-efficacy, and action control in the adoption and maintenance of physical exercise. Psychol. Health.

[94] Padgett D. (2000). “Americans need something to sit on,” or Zen meditation materials and Buddhist diversity in North America. J. Glob. Buddhism.

[95] Prebish C.S., Baumann M. (2013). Westward Dharma: Buddhism beyond Asia. Choice Rev. Online.

[96] Ajzen I. (1991). The theory of planned behavior. Organ. Behav. Hum. Decis. Process..

[97] Morwitz V.G., Fitzsimons G.J. (2004). The Mere-Measurement Effect: Why Does Measuring Intentions Change Actual Behavior?. J. Consum. Psychol..

